# Gabapentinoid prescriptions for neuropathic and musculoskeletal pain in Lebanon

**DOI:** 10.2144/fsoa-2023-0219

**Published:** 2024-05-14

**Authors:** Joe Rassi, Stephanie Khazaka, Sani Hlais, Stephanie Rassi, Mohammad Daher, Toufic Samaha

**Affiliations:** 1 Orthopedic Department, Hotel Dieu de France, Beirut, Lebanon; 2 Saint Joseph University, Beirut, Lebanon; 3 Orthopedics department, Brown University, Providence, RI, 02906

## Abstract

**Aim:** The purpose of this study is to analyze the different characteristics of gabapentinoids prescription by Lebanese orthopedics surgeons. **Methods:** This is an observational, cross-sectional study using a survey which was carried out in collaboration with the Lebanese Orthopedic Society over a 3-month period. **Results:** Forty-two orthopedic surgeons responded, most of them prescribing gabapentinoids in their daily practice with only half of the patients feeling relief after taking them. Furthermore, most of the surgeons prescribed these drugs for patients above 18 years old and for both acute and chronic pain. **Conclusion:** Even though almost half of the patients do not experience relief after taking gabapentinoids, these drugs are becoming more and more prescribed.

## Plain language summary

With pain being a major concern in orthopedic surgery, a lot of surgeons prescribe drugs before surgical procedures for their patients. One of these drugs categories include Gabapentinoids. This class of medications was shown to be prescribed more and more by orthopedic surgeons despite it not granting successful pain relief for half of the patients and not being US FDA approved.

## Abstract (plain language summary)

97.62% of surveyed orthopedic physicians prescribe gabapentinoids in daily practice with neuropathic and musculoskeletal pain being the main indication.

83% of surveyed surgeons use gabapentinoids when initial treatment for orthopedic pain fails with pregabalin (PGB) being prescribed slightly more (51.2%) than gabapentin (GBP) (48.8%).

Minimum effective dose: 300 mg for GBP (70.73%), 75 mg for PGB (66.67%) with common side effects: dizziness (80.95%), somnolence (76.19%), visual disturbances (30.95%) being unrelated to the dosage.

97.62% prescribe gabapentinoids with other analgesics, and rarely as monotherapy (2.38%).

Criteria influencing choice: type of pain (67.5%), safety and side-effect profile (57.5%) with age not considered a limiting factor by 63.41% of surveyed surgeons.

Despite nearly half of the individuals not finding relief with gabapentinoids, the prescription of these medications is on the rise.

Orthopedic consultations include patients suffering from pain, bone misalignments, abnormal growth or development, loss of mobility or patients requiring orthopedic surgery [1]. Pain is the most common symptom of most musculoskeletal conditions [^2-7^]. There are inter- and intra-individual variations [2]. An individual may experience pain that is local or diffuse, acute or chronic, of short or long duration and from mild to severe [1,7].

Gabapentinoids, existing as Gabapentin (GBP), or Pregabalin (PGB) are antiepileptic drugs that can be as well used for the management of pain. In fact, the Food and Drug Administration (FDA)-approved GBP for post-herpetic neuralgia, restless leg syndrome and convulsions [8]. As for PGB, it was approved for neuropathic pain (including post-herpetic neuralgia), fibromyalgia and convulsions [9]. However, gabapentinoids prescriptions have significantly increased in recent years in the management of non-FDA-approved musculoskeletal conditions [10,11].

Therefore, the purpose of this study is to identify the minimum effective daily dose of gabapentinoids prescribed by Lebanese orthopedists as well as their main indications, evaluate the influence of side effects on the choice of this dose, and identify the factors that influence the choice of one of these molecules to treat neuropathic and musculoskeletal pain in Lebanese patients.

## Materials & methods

This is an observational, cross-sectional study using a survey for data collection. The survey was carried out in collaboration with the Lebanese Orthopedic Society (LOS) over a 3-month period. This questionnaire (cf. Supplementary annex 1) was sent by e-mail to orthopedic surgeons. The LOS sent it on several occasions to all doctors belonging to this society. Participants were briefed on the purpose of the survey.

### Data

The data collected from the questionnaire concerns the prescription of gabapentinoids, the different indications (in orthopedics), the type of pain (acute or chronic), when gabapentinoids are prescribed (as a first-line treatment or not), the age required for prescription, criteria influencing the choice of one of the molecules (pregabalin or gabapentin), i.e., side effects, dosage, price, prescriptions of gabapentinoids alone or in combination with other analgesics, and the percentage of patients satisfied with this analgesic treatment.

### Ethical considerations

The agreement of the local ethical committee and that of the LOS were obtained prior to conducting the study. Data entry and analysis were carried out while respecting the anonymity and confidentiality of the participants.

### Statistics

The data have been analyzed using the SPSS 26.0 software (SPSS Inc., IL, USA). Categorical variables were described in terms of numbers and percentages. Associations were tested using the Chi-2 test for percentage comparisons, and the Fisher test for small numbers. The alpha significance level was set at 5%.

## Results

### Use, preferences & indications of gabapentinoids in orthopedics

**Figure 1.**
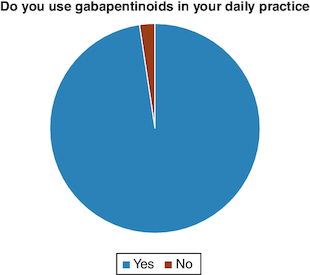
Pie-chart showing the percentage of utilization of gabapentinoids by Lebanese orthopedic surgeons.

**Figure 2.**
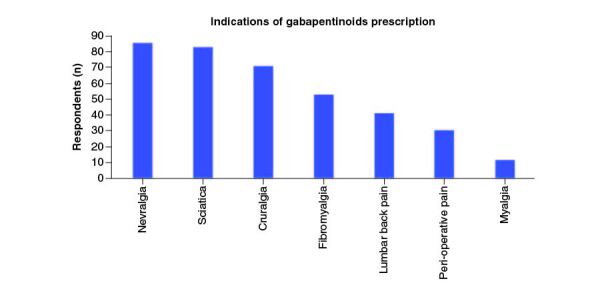
Plot showing the percentage of prescription of gabapentinoids for each indication.

**Figure 3.**
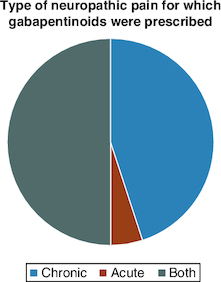
Pie-chart showing the types of neuropathic pain for which gabapentinoids were prescribed.

**Figure 4.**
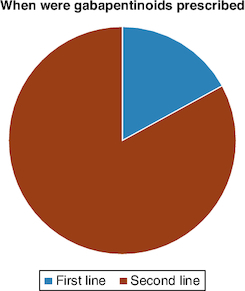
Pie-chart showing the percentage of surgeons prescribing gabapentinoids as a first and second line.

Of the 42 orthopedic physicians who responded to the questionnaire, 41 (97.62%) prescribe gabapentinoids in their daily practice, and only one (2.38%) does not (Figure 1). The main indication for all doctors (100%) was neuropathic and musculoskeletal pain. The types of pain for which gabapentinoids were most prescribed were neuropathic pain: neuralgia (85.37%), sciatica (82.93%) and cruralgia (70.73%) (Figure 2). Half of doctors prescribed gabapentinoids for both acute and chronic pain, 45% for chronic pain only, and 5% for acute pain only (Figure 3). For orthopedic pain, around 83% of doctors prescribed gabapentinoids when initial treatment had failed, while 17% prescribed them as first-line treatment (Figure 4).

The questionnaire also revealed that 47.5% of doctors prescribed gabapentinoids for the 18–65 age group; 42.5% for the >18 age group; 7.5% for all age groups and only 2.5% for the >65 age group.

Of the two molecules (PGB and GBP), the tendency to prescribe PGB (51.2%) was slightly higher than for GBP (48.8%). The majority of orthopedic physicians (61.9%) felt that these side effects were more likely to be encountered with PGB (73.08%) than with GBP (26.92%). Furthermore, 61.9% of physicians felt that some patients were better able to tolerate one molecule than the other.

### Efficacy & influence of side effects on the choice of minimum dose

**Figure 5.**
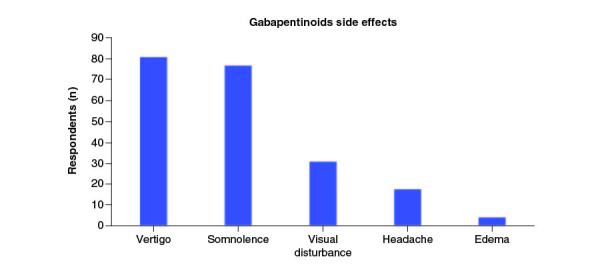
Plot showing the percentage of gabapentinoids side effects experienced by the patients.

According to the study, the minimum effective dose with which orthopaedic surgeons tended to start treatment was 300 mg for GBP (70.73%), and 75 mg for PGB (66.67%). Dizziness (80.95%), somnolence (76.19%) and visual disturbances (30.95%) were the most common side effects encountered during treatment (Figure 5).

To determine whether there was a correlation between dose and side effect, the two variables ‘minimum effective dose of GBP’ and ‘minimum effective dose of PGB’ were dichotomized: the lowest GBP dose (300 mg) was compared with the other doses (400; 600; 900 mg) for the occurrence of the side effects: vertigo, somnolence, edema, headache and visual disturbances; and the 25–50 mg PGB doses were compared with those of 75–150 mg for the occurrence of the same side effects already mentioned. It was shown that the dosage of GBP and PGB were not related to vertigo (p = 1.000 and p = 0.336, respectively), somnolence (p = 1.000 and p = 0.660, respectively), edema (p = 0.505 and p = 1.000, respectively), headache (p = 1.000 and p = 0.631, respectively) and visual disturbance (p = 0.285 and p = 0.422, respectively).

Almost all orthopedic physicians prescribe gabapentinoids in combination with other analgesics (97.62%), rarely as monotherapy (2.38%) with the majority of gabapentinoids being prescribed for an average duration of 1–3 months (80.49%). The percentage of patients relieved by gabapentinoid treatment for the majority of physicians was 50–75% (45.24%).

### Factors influencing choice of molecule

#### Age

The majority of doctors (i.e. 63.41%) did not consider age to be a limiting factor in prescribing gabapentinoids, while 36.59% did.

#### Other criteria influencing choice

The criteria that most influenced the choice of treatment were, firstly, the type of pain (67.5%), then the safety and side-effect profile (57.5%), pharmacokinetic properties (30%), the presence of comorbidities (20%) and, lastly, cost (10%).

A statistical analysis was carried out to determine whether there was a correlation between the choice of one molecule or the other and age, type of pain, safety profile, pharmacokinetic properties, comorbidities and cost. No significant associations were found for any of these variables. Furthermore, the results showed no significant association between molecule preference and the occurrence of side effects: dizziness, somnolence, edema, headache and visual disturbances. Likewise, there was no significant association between molecule preference and the impression that side effects are the prerogative of one molecule more than another, tolerance, time of prescription, duration and relief. Finally, there was no significant correlation between the choice of one of the two molecules and the type of pain (myalgia, sciatica, fibromyalgia, neuralgia, lumbago, cruralgia, perioperative pain), nor with the acute or chronic nature of the pain.

## Discussion

The FDA has approved the use of GBP and PGB for three indications: epileptic seizures, postherpetic or diabetic neuropathic pain and fibromyalgia (only for PGB). However, prescriptions for gabapentinoids have increased remarkably in recent years, in connection with many other indications, as our study shows, although these are not yet approved by the FDA. Baftiu *et al.* report that antiepileptic drugs such as gabapentinoids are now being used for indications other than epilepsy [11]. Furthermore, an estimate by Anantharamu *et al.* suggests that over 95% of gabapentinoid prescriptions are used outside the three approved indications [10]. In the USA, prescriptions for these molecules have more than tripled in the last 10 years, as described by Johansen *et al.* and Montastruc *et al.* [12,13]. Although, the explanation for this significant increase is not really clear, some doctors, such as Rees *et al.* and Enke *et al.*, justify it by the search for opioids and NSAIDs alternatives because of their side effects [14,15]. Goodman *et al.* advise physicians on the minimal evidence of efficacy for non-approved indications, and insist on warning patients that the benefits are not yet certain [16]. Despite this, orthopedic physicians continue to prescribe them for various types of pain (neuralgia, sciatica, cruralgia, low back pain, fibromyalgia, myalgia and peri-operative pain). It should be noted that GBP has not yet been approved for the treatment of fibromyalgia, and peri-operative pain is a recent indication in which gabapentinoids have yet to prove their place and benefit. Furthermore, Fabritius *et al.* do not recommend their systemic use for postoperative pain [17]. However, as our results indicate, doctors prescribe gabapentinoids for chronic pain rather than acute pain, which is in line with recommendations in the literature. Moreover, the majority of the Lebanese orthopedic physicians do not recommend gabapentinoids as a first-line treatment, which is in line with recent major guidelines requiring the use of gabapentinoids as a second-line treatment if antidepressants were ineffective in neuropathic pain [18,19].

When it comes to the prescription age of gabapentinoids for pain, it is adults and the elderly who benefit most. Indeed, a small percentage of doctors prescribe these molecules for all age groups, while the majority of other doctors recommend them from the age of 18 upwards. This may be consistent with the low number of clinical trials in children and adolescents, as explained by Egunsola *et al.* compared with the high number of trials in adults and even the elderly [20]. In addition, a systematic review by Chen *et al.* about the usage of gabapentinoids in pediatric patients was not able to recommend the routine usage of these drugs due to the lack of data regarding this matter [21]. Johanson *et al.* report that it is the elderly with comorbidities who use and benefit from these molecules, since they are not metabolized by the liver (low risk of interactions with other concomitant treatments), provided their renal function is taken into account (need for dosage adjustment) and they are warned of some possible side effects [12]. Furthermore, in a similar survey, Girdler *et al.* reported gabapentin to be the most common medication prescribed pre-operatively in patients with adolescent idiopathic scoliosis [22].

As for the choice between the two gabapentinoid molecules, few clinical trials have been carried out to compare the two molecules with each other, as noted by Calandre *et al.* [23]. Moreover, as our results have shown, there are no factors that would favor one molecule over the other. This was in line with the findings in the literature stating that the choice is independent of age, type of pain, the safety profile or side effects, pharmacokinetic properties, the presence of comorbidities, or cost [16,^24-26^]. In addition, Shanthanna *et al.* found that after two consecutive years, the percentages of increase in PGB (53%) and GBP (46%) were very close, which is in agreement with our results (51 vs 48%) [27]. As for the choice of the first minimum daily dose in pain management, it is recommended to start PGB with a dose of 75 or 150 mg as mentioned by Bockbrader *et al.* and Calandre *et al.*, and GBP with a dose of 300 mg/d as also reported by Bockbrader *et al.* and Backonja *et al.* [23,24,28]. Our results are in line with these recommendations.

Gabapentinoids are generally administered in combination with other analgesics. Our results confirm this (97% in combination and 3% as monotherapy). In fact, the concomitant use of several analgesics with different mechanisms of action means lower doses of each drug (and therefore fewer side effects) and better patient analgesia than with higher doses of a single drug. However, not all analgesic combinations are ideal. Some may potentiate the side effects of combined drugs, despite lower doses and better analgesia. This is, for example, the case with gabapentinoids combined with opioids, as clearly demonstrated by Kharasch *et al.* with regard to the increase in respiratory depression, especially in postoperative analgesia, and Jongen *et al.* who recommended multimodal analgesic prescription, taking into consideration the medical profile of each patient [29,30]. Furthermore, Giakas *et al.* reported that using gabapentin post-operatively reduced opioid consumption in patients in patients undergoing sports medicine surgeries while providing a similar pain control [31]. Similar findings were shown by Zhang *et al.* in patients undergoing posterior lumbar fusion [32]. However, gabapentin did not have an impact of length of stay which is an important consideration in spine surgery [33].

As for the duration of treatment with gabapentinoids, it remains unknown in the literature. However, our results show that between one and three months are required for optimal analgesic treatment. When studying the percentage of satisfied patients after treatment with gabapentinoids, only half of those treated with these molecules are relieved of their pain. Even according to a review carried out by the Canadian Agency for Drugs and Technologies in Health, only 13–38% of patients report pain relief [34]. This suggests the need for further clinical studies to confirm the scope, indications and treatment duration of gabapentinoids [16,35].

## Conclusion

Most of the gabapentinoids prescription terms by Lebanese orthopedic surgeons are in line with the recommendations except for the indications. Therefore, more clinical data on gabapentinoids is needed to better manage non-FDA-approved indications. There is also a need for a better understanding of pain and its causes, in order to bring relief to as many patients as possible. Many more prospective studies are needed to confirm the efficacy of gabapentinoids in non-approved indications, and to avoid over-prescribing gabapentinoids.

## Supplementary Material

Supplementary Annex 1
