# Supplementary material for: Gabapentinoid prescriptions for neuropathic and musculoskeletal pain in Lebanon
Source: Future Sci OA. 2024 May 14;10(1):FSO960. doi: 10.2144/fsoa-2023-0219 (PMC11137850; doi:10.2144/fsoa-2023-0219)
Supplement: Supplementary Annex 1 [file IFSO_A_2340257_SM0001.docx]

QUESTION 1: Do you prescribe gabapentinoids in your daily practice?

- Yes

- No

QUESTION 2: What is the main indication?

- Neuropathic and musculoskeletal pain

- Other indications

Specify: ..............................................................................................

QUESTION 3: In what type of pain would you prescribe gabapentinoids?

- Myalgia

- Sciatica

- Fibromyalgia

- Neuralgia

- Low back pain

- Cruralgia

- Peri-operative pain

QUESTION 4: For which neuropathic pain would you prescribe gabapentinoids?

- Acute

- Chronic

QUESTION 5: For orthopaedic pain, do you prescribe gabapentinoids as a?

- First-line treatment

- Failure of initial treatment

QUESTION 6: Does age limit your prescription of these molecules?

- Yes

- No

QUESTION 7: For which age group(s) do you prescribe gabapentinoids?

- < 18 years

- 18 - 65 years

- > 65 years

QUESTION 8: Which of the two gabapentinoid molecules do you tend to prescribe

prescribe as first-line treatment?

- Gabapentin

- Pregabalin

QUESTION 9: Which criteria most influences the choice of treatment?

-Type of pain.

- Safety and side-effect profile

- Cost

- Pharmacokinetic properties (speed of action, bioavailability)

- Presence of comorbidities (drug interactions)

QUESTION 10: What are the most common side effects of treatment you encounter?

- Dizziness

- Drowsiness

- Edema

- headache

- visual disturbances

QUESTION 11: Do you have the impression that these side effects are the prerogative of one compound more than another?

- No

- Yes

If yes, which one?

- Gabapentin

- Pregabalin

Question 12: Do you have the impression that some patients are better able to tolerate one than the other molecule?

- No

- Yes

QUESTION 13: In your experience, what is the minimum effective daily dose with which with which you tend to start your treatment?

a) Gabapentin :

- 300mg

- 400mg

- 600mg

- more

b) Pregabalin:

- 25mg

- 50mg

- 75mg

- 150mg

- more

QUESTION 14: Do you prescribe gabapentinoids as monotherapy or in combination

with other treatments?

- Monotherapy

- Combination with other analgesics

QUESTION 15: Do you prescribe gabapentinoids for an average duration of :

- < 1 month

- 1-3 months

- > 3 months

QUESTION 16: In your experience, what percentage of patients feel relieved by this treatment?

- < 25%

- 25-50%

- 50-75%

- > 75%
